# The battle to save the anus: a triumph of careful colonoscopy and medical history taking

**DOI:** 10.1055/a-2410-3776

**Published:** 2024-09-25

**Authors:** Huankai Shou, Lina Fan, Xinyang Liu, Xian-Li Cai, Ping-Hong Zhou

**Affiliations:** 1 92323 Endoscopy Center and Endoscopy Research Institute, Zhongshan Hospital Fudan University, Shanghai, China; 2 92323 Nursing Department, Zhongshan Hospital Fudan University, Shanghai, China

A 60-year-old woman attended the endoscopy center for a colonoscopy and biopsy of a suspected rectal cancer. The patient had been suffering from fever and abdominal pain for 10 days, with a history of hematochezia and constipation. The local hospitalʼs computed tomography (CT) scan had suggested thickening of the rectal wall with small lymph nodes, and that malignancy could not be ruled out. Initial colonoscopy at the local hospital had revealed a suspicious mass with an ulcer in the rectum, giving a high suspicion of malignancy ( Fig. 1 **a** ). The biopsy showed focal lymphocytic proliferation, and rebiopsy, with multiple and larger samples, was recommended. A second colonoscopy was therefore performed. Unexpectedly, a long sinus with pus was revealed once the rectum was sufficiently inflated, which had not been found during the first colonoscopy ( Fig. 1 **b** ). The sinus extended from the external orifice on the mucosal layer to the submucosal layer, where the muscular layer remained intact ( Fig. 1 **c** ). These findings did not suggest rectal cancer, instead they appeared more consistent with an infected submucosal tunnel. But why was there such a sinus?

**Fig. 1:**
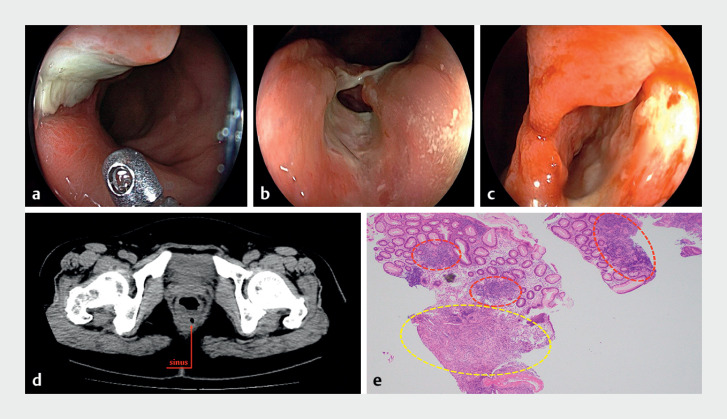
Colonoscopic appearance of the rectal mass at: **a** the first colonoscopy, showing a mass with an ulcer in the rectum, with a high suspicion of malignancy; **b, c** the second colonoscopy showing: **b** a long sinus with pus; **c** the sinus extending from the external orifice on the mucosal layer to the submucosal layer, where the muscular layer remained intact. **d** Repeat computed tomography scan showing a sinus about 1.5–2 cm in the submucosal layer of the rectum. **e** Further pathologic sample showing focal lymphocytic infiltration (red circles) and inflammatory granulation tissue (yellow circle), consistent with inflammation without any evidence of malignancy.

Usually, sinus tracts are caused by surgery, inflammatory bowel disease, infection, trauma, and tumors, among other reasons. Consequently, an in-depth meticulous medical history was obtained to elucidate the potential underlying cause, revealing that, 10 days previously, the patient, who had been struggling with severe constipation, had had a glycerin suppository administered and had subsequently experienced the onset of her symptoms, with locally performed blood examination showing a white cell count (WBC) of 13.30 × 109/L and C-reactive protein (CRP) of 57 mg/L. Based on these findings, the patient was referred to the emergency room for comprehensive evaluation, including a repeat CT scan and blood examination, with the surgeon being informed of the endoscopic findings, along with the medical history. The second CT suggested a sinus about 1.5–2 cm in the submucosal layer of the rectum, correlating with the endoscopic findings ( Fig. 1 **d** ). Blood examination revealed a WBC of 7.90 × 109/L and CRP of 5 mg/L. A second pathologic examination indicated inflammation without any evidence of malignancy ( Fig. 1 **e** ). Ultimately, anti-inflammatory treatment was given and the patient reported no abdominal pain or fever during subsequent outpatient follow-up. The culprit behind this rectal mass was not a cancer, but a glycerin suppository; the battle to save the anus was a success ( Video 1 ).

A careful colonoscopy and medical history-taking saved the patientʼs anus in a suspected rectal malignancy that turned out to be an inflammatory sinus related to administration of a suppository.Video 1

Although rectal injuries are relatively rare, they can be difficult to diagnosis and are often missed at patients’ initial presentation 1 . Sometimes it is challenging to distinguish them from tumors, especially when the patient has relatively common “alarm” symptoms of rectal cancer, such as hematochezia and change in bowel habit 2 . The role of the medical history cannot not be overemphasized, and we should take a comprehensive view of the patientʼs medical history in order to obtain a more accurate diagnosis.

Endoscopy_UCTN_Code_TTT_1AQ_2AB

## Notes

Endoscopy E-Videos https://eref.thieme.de/e-videos
*E-Videos* is an open access online section of the journal *Endoscopy* , reporting on interesting cases and new techniques in gastroenterological endoscopy. All papers include a high-quality video and are published with a Creative Commons CC-BY license. Endoscopy E-Videos qualify for HINARI discounts and waivers and eligibility is automatically checked during the submission process. We grant 100% waivers to articles whose corresponding authors are based in Group A countries and 50% waivers to those who are based in Group B countries as classified by Research4Life (see: https://www.research4life.org/access/eligibility/ ). This section has its own submission website at https://mc.manuscriptcentral.com/e-videos .
